# On the evaluation of mobile target trajectory between four‐dimensional computer tomography and four‐dimensional cone‐beam computer tomography

**DOI:** 10.1002/acm2.13310

**Published:** 2021-06-03

**Authors:** Colton Baley, Neil Kirby, Timothy Wagner, Nikos Papanikolaou, Pamela Myers, Karl Rasmussen, Sotirios Stathakis, Daniel Saenz

**Affiliations:** ^1^ Department of Radiation Oncology School of Medicine The University of Texas Health Science Center at San Antonio San Antonio TX USA

**Keywords:** 4D CBCT, 4D CT, lung cancer, motion management, SBRT

## Abstract

**Purpose:**

For mobile lung tumors, four‐dimensional computer tomography (4D CT) is often used for simulation and treatment planning. Localization accuracy remains a challenge in lung stereotactic body radiation therapy (SBRT) treatments. An attractive image guidance method to increase localization accuracy is 4D cone‐beam CT (CBCT) as it allows for visualization of tumor motion with reduced motion artifacts. However, acquisition and reconstruction of 4D CBCT differ from that of 4D CT. This study evaluates the discrepancies between the reconstructed motion of 4D CBCT and 4D CT imaging over a wide range of sine target motion parameters and patient waveforms.

**Methods:**

A thorax motion phantom was used to examine 24 sine motions with varying amplitudes and cycle times and seven patient waveforms. Each programmed motion was imaged using 4D CT and 4D CBCT. The images were processed to auto segment the target. For sine motion, the target centroid at each phase was fitted to a sinusoidal curve to evaluate equivalence in amplitude between the two imaging modalities. The patient waveform motion was evaluated based on the average 4D data sets.

**Results:**

The mean difference and root‐mean‐square‐error between the two modalities for sine motion were −0.35 ± 0.22 and 0.60 mm, respectively, with 4D CBCT slightly overestimating amplitude compared with 4D CT. The two imaging methods were determined to be significantly equivalent within ±1 mm based on two one‐sided *t* tests (*p* < 0.001). For patient‐specific motion, the mean difference was 1.5 ± 2.1 (0.8 ± 0.6 without outlier), 0.4 ± 0.3, and 0.8 ± 0.6 mm for superior/inferior (SI), anterior/posterior (AP), and left/right (LR), respectively.

**Conclusion:**

In cases where 4D CT is used to image mobile tumors, 4D CBCT is an attractive localization method due to its assessment of motion with respect to 4D CT, particularly for lung SBRT treatments where accuracy is paramount.

## 1 INTRODUCTION

Since the introduction of image‐guided radiation therapy (IGRT), many imaging modalities have been employed to increase the accuracy at which mobile lung tumors are treated. These modalities include megavoltage (MV) cone‐beam computed tomography (CBCT), kilovoltage (kV) CBCT, tomotherapy MV computed tomography (CT), and room mounted kV planar imaging with or without fiducials.^1^, ^2^, ^3^, ^4^, ^5^ Given that lung cancer is the leading cause of cancer death in the United States as reported by the National Cancer Institute,^6^ improving treatment techniques is imperative. A technique that was developed most recently incorporates a time element into the on‐board CBCT of most modern linear accelerators (LINACs). This technique allows the user to create 4D data sets from reconstruction of phase‐correlated planar kV images and is known as 4D CBCT. The images used to construct the 4D CBCT are obtained by acquiring a CBCT over 200° in a time frame of approximately 4 min.^7^ The 4D CBCT protocol allows for the entire respiratory cycle to be imaged over very small ranges in angle during the gantry rotation, a process known as oversampling. The oversampled images are then binned into different phases of the respiratory cycle. Determining where an image is binned in the respiratory cycle is based on the position of high‐contrast anatomical landmarks such as the diaphragm in each image.^8^, ^9^, ^10^ After the images are binned, a 3D CBCT is reconstructed for each phase of the respiratory cycle, where the typical number of phases is 10. The 4D CBCT allows for accurate visualization of the tumor in each phase of the respiratory cycle by reducing motion artifacts associated with traditional 3D CBCT imaging acquisitions.

The accuracy at which targets may be localized from utilization of the 4D CBCT image acquisition makes it an appealing candidate for lung stereotactic body radiation therapy (SBRT) image guidance. With SBRT, the dose per fraction is much higher than that used in conventional radiotherapy treatments, and far fewer fractions are used, leading to a more potent biological effect.^11^, ^12^ Because the dose per fraction is high, dose gradients must be steep to minimize unnecessary toxicity to normal tissues surrounding the treatment site, and target localization must be very accurate to ensure no geographic miss. With respect to target localization, 4D CBCT has been shown to better localize mobile lung tumors while maintaining comparable image quality when compared with traditional 3D CBCT image guidance techniques used in lung treatment protocols.^7^, ^13^ Application of SBRT to certain lung cancers has been shown to be as effective as conventional radiation therapy with the added benefit of patient convenience in much shorter treatment times.^14^, ^15^ It has also become a viable alternative to surgically inoperable patients and those with oligometastatic disease.^16^, ^17^ By increasing the confidence of localization with 4D CBCT, these outcomes may further improve.

To recognize the benefits 4D CBCT may provide, it is important to understand how 4D CBCT target trajectories compare with those at simulation. Often, patients are simulated with a 4D CT image acquisition. The 4D CT is employed to learn about the target’s path and excursion and allow for accurate delineation of the mobile target. Because the treatment plan is based upon the motion at simulation, it is important to establish how the target trajectory acquired from the 4D CT compares with that during treatment of the 4D CBCT to ensure proper dose coverage. Previously, differences in 4D CT and 4D CBCT evaluation of target motion for patient respiratory patterns have been explored.^18^, ^19^, ^20^ These studies have found potential discrepancies between the two imaging modalities in the presence of patient respiration where breathing irregularity is common. It is challenging though to determine if these discrepancies are due to patient‐related factors or inherent differences in the imaging modalities. Additionally, 4D CT has been shown to suffer from motion artifacts leading to 4D CBCT better evaluating target volumes when using a motion phantom with a few different sinusoidal and patient specific motion parameters.^21^ Lastly, phantom target motion between the two imaging modalities has been shown to be comparable but with very few different motion parameters explored.^22^ These studies, however, have not explored how a wide variety of combinations of amplitude and cycle time for the target motion affect the agreement between 4D CT and 4D CBCT evaluation of target trajectories and where evaluation is hindered due to artifacts. Additionally, it is unclear if previously studied discrepancies in target motion evaluation are inherent to the two modalities or due to patient specific factors such as breathing irregularity. This study poses two aims, (a) to investigate several combinations of target amplitude and cycle time on a dynamic thorax phantom using sine motion to remove the variability of patient respiration and evaluating if the two modalities are equivalent within a given difference interval and (b) evaluate the motion measured using previous patient breathing waveforms to simulate patient breathing.

## 2 MATERIALS AND METHODS

### 2.1 Phantom motion

**Fig. 1:**
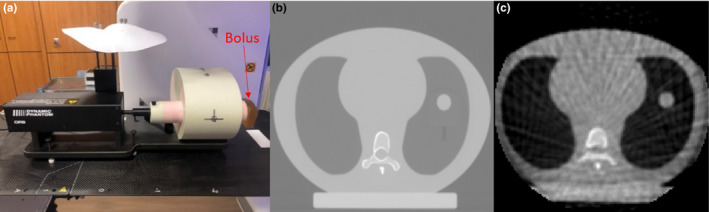
Illustration of the CIRS dynamic thorax phantom setup with bolus (a) with axial four‐dimensional computer tomography (4D CT) (b) and four‐dimensional cone‐beam computer tomography (4D CBCT) (c) slices demonstrating the phantom geometry.

To perform this study, the Computerized Imaging Reference Systems (CIRS, Norfolk, VA) dynamic thorax phantom model 008A was employed. Figure 1 shows the phantom used in this study, with 4D CT and 4D CBCT axial slices demonstrating the phantom’s geometry. The Trio PC Motion library was used to alter the motion parameters of the phantom.

### 2.2 Sine motion

The imaging rod with a 2‐cm diameter soft tissue equivalent target was selected. The sine motion signal was used with cycle times and amplitudes chosen based on practical respiratory rates and previously observed motion amplitudes from a large sample of lung cancer patients.^23^, ^24^ This resulted in amplitudes in the superior–inferior (SI) directions of 3, 5, 7, 9, 11, 13, 15, 17, and 19 mm. Each parameter was paired with cycle times of 3, 5, and 7 s except for 3, 5, 17, and 19 mm.

### 2.3 Patient waveforms

**Fig. 2:**
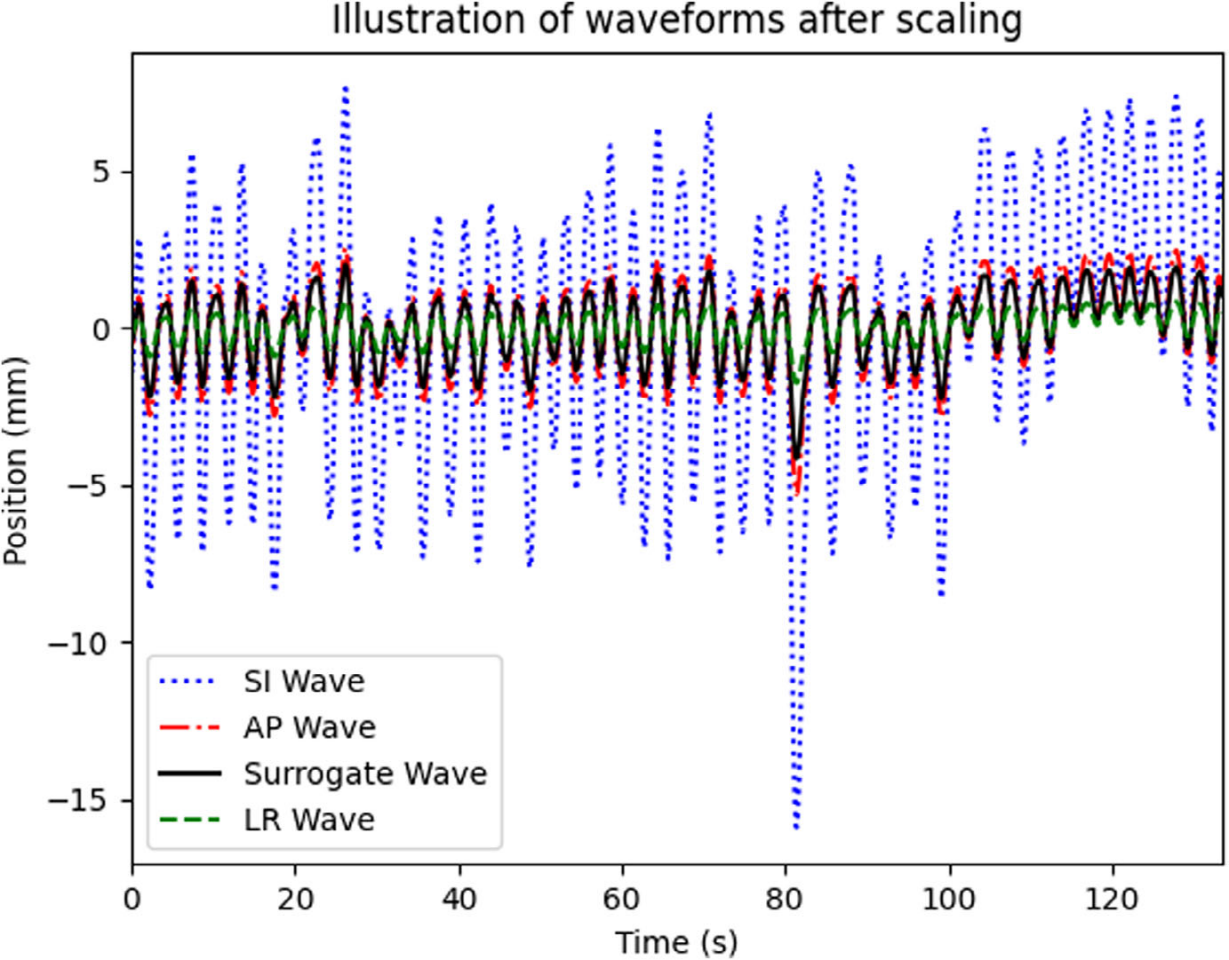
Example of surrogate trace scaled to generate patient‐specific tumor motion in all three directions.

Surrogate amplitude traces from seven previous patients were exported to generate patient‐specific waveforms. The amplitude traces were imported into python and smoothed using the Savitzky–Golay filter. Peaks and troughs were then computed to determine the difference between the average peak and the average trough value. This value was used to scale patient waveforms to that of tumor motion measured in GE Advantage 4D in the SI, anterior–posterior (AP), and left–right (LR) directions. This process was repeated for each patient until a scaled waveform was generated for each direction (SI, AP, and LR). Data were then imported into the phantom controller software to drive the motion for all three directions and the surrogate. An example of a patient‐specific waveform for each direction is shown in Fig. 2. Phantom target size was selected based on the volume closest to the patient’s tumor size and was chosen from fixed diameters of 1, 2, or 3 cm.

### 2.4 4D CT acquisition

The process used for 4D CT simulation of lung SBRT patients at our institution was followed. The 4D CT data sets were acquired with a cine scan using a GE lightspeed 16‐slice CT scanner with a rotation time of 1 s, slice thickness of 2.5 mm, and pixel spacing of 1.27 mm. Surface imaging was used as a marker of respiratory phase for retrospective 4D binning using CRAD (Uppsala, Sweden) Sentinel. A region of interest was set on the surface of the surrogate of the CIRS Motion phantom to track the simulated respiratory cycle. To ensure an entire respiratory cycle was captured at each table position, the CT scanner cine time was set to include the cycle time plus 1.5 s. This is consistent with clinical practice where the additional time margin ensures full capture of the respiratory cycle in the presence of variable respiratory rates. Once the 4D CT data were acquired, it was exported and reconstructed using GE Advantage 4D (GE Healthcare, Chicago, IL). All 10 phase CT image sets were exported for analysis.

### 2.5 4D CBCT acquisition

Elekta’s kV CBCT system (XVI) was used on the VersaHD LINAC to obtain the 4D CBCT data sets employing the Symmetry imaging protocol (Elekta, Stockholm, Sweden). A high‐contrast object is required to accurately bin the images into the appropriate respiratory phase. A bolus was added to the end of the imaging rod acting as a high‐contrast object to reduce discrepancies in phase sorting as shown by Liang et al.^8^ The configuration of the added bolus on the imaging rod is shown Fig. 1. The 4D CBCT scanning protocol consisted of the acquisition of 975 frames (20 mA and 16 ms per frame) over a 200° counterclockwise arc of gantry rotation. Elekta’s F0 filter and S20 collimators were used. Images were reconstructed with a nominal slice thickness and pixel spacing of 1 mm. All phases from the 4D CBCT were DICOM exported through MOSAIQ to be processed offline.

### 2.6 Calculating trajectories

#### 2.6.1 Sine motion

Determining target trajectory throughout the different phases of respiration was conducted with an in‐house python code to process the images. Phase images were imported and organized into a 4D matrix then converted to binary images using the Otsu thresholding method.^25^ Erosion followed by dilation was applied in some instances to remove noise‐based objects. After image processing, the approximate coordinates of the target centroid were selected using a built‐in slice viewer. The binary data set was then labeled to find each object in the data set. The target object was determined by minimizing the Euclidean distance between the previously selected coordinates that approximated the centroid using the slice viewer and the centroid of each labeled object. Image processing and labeling was done almost exclusively using the skimage library.^26^ The coordinates and centroid of the concluded object depicting the target were stored to segment the target and for centroid determination and curve fitting.

Sinusoidal curve fitting of each phase’s centroid was used to measure the motion amplitude. Segmentation was employed to visually inspect if the target was properly found in each phase using the same slice viewer in the axial and coronal planes. Data were fit using SciPy library’s least squares with an optimization function minimizing the difference between the variable sine function parameters (amplitude, frequency, phase shift, and vertical shift) and the data points of the centroid position for each respiratory phase.^27^ Curve fitted amplitudes were then compared between the two imaging modalities. Additionally, a second method of amplitude evaluation was employed because curve fitting was unacceptable in the presence of motion artifacts in a few motion combinations. This method used the average CT of the 4D CT data set with a window level and width of −400 and 1500, respectively, to measure amplitude through visual inspection.

The concluded curve fitted amplitudes were evaluated to determine equivalence between the two imaging modalities. Equivalence was tested by the two one‐sided *t*‐test (TOST) method.^28^, ^29^ The upper and lower limits used for the TOST were 1 and −1 mm, respectively. The 1‐mm range was chosen due to the setup error considered acceptable in SBRT treatments.^30^ Additionally, coefficients of correlation and determination between the true amplitude programmed and both imaging methods were employed.

#### 2.6.2 Patient waveforms

Target motion evaluation for simulated patient respiratory motion scenarios was based on average 4D CT and 4D CBCT data sets. Individual phase data were not used due to many phases being too noisy or containing artifacts due to the irregularity between cycles. Data sets were imported into python and interpolated to allow auto segmentation to 0.1 mm of accuracy. The interpolated data sets were binarized based on manual threshold values representing the most visually appropriate contour. The dimensions of these contours were used to determine the amplitudes in the SI, AP, and LR directions.

## 3 RESULTS

### 3.1 Sine motion

**Fig. 3:**
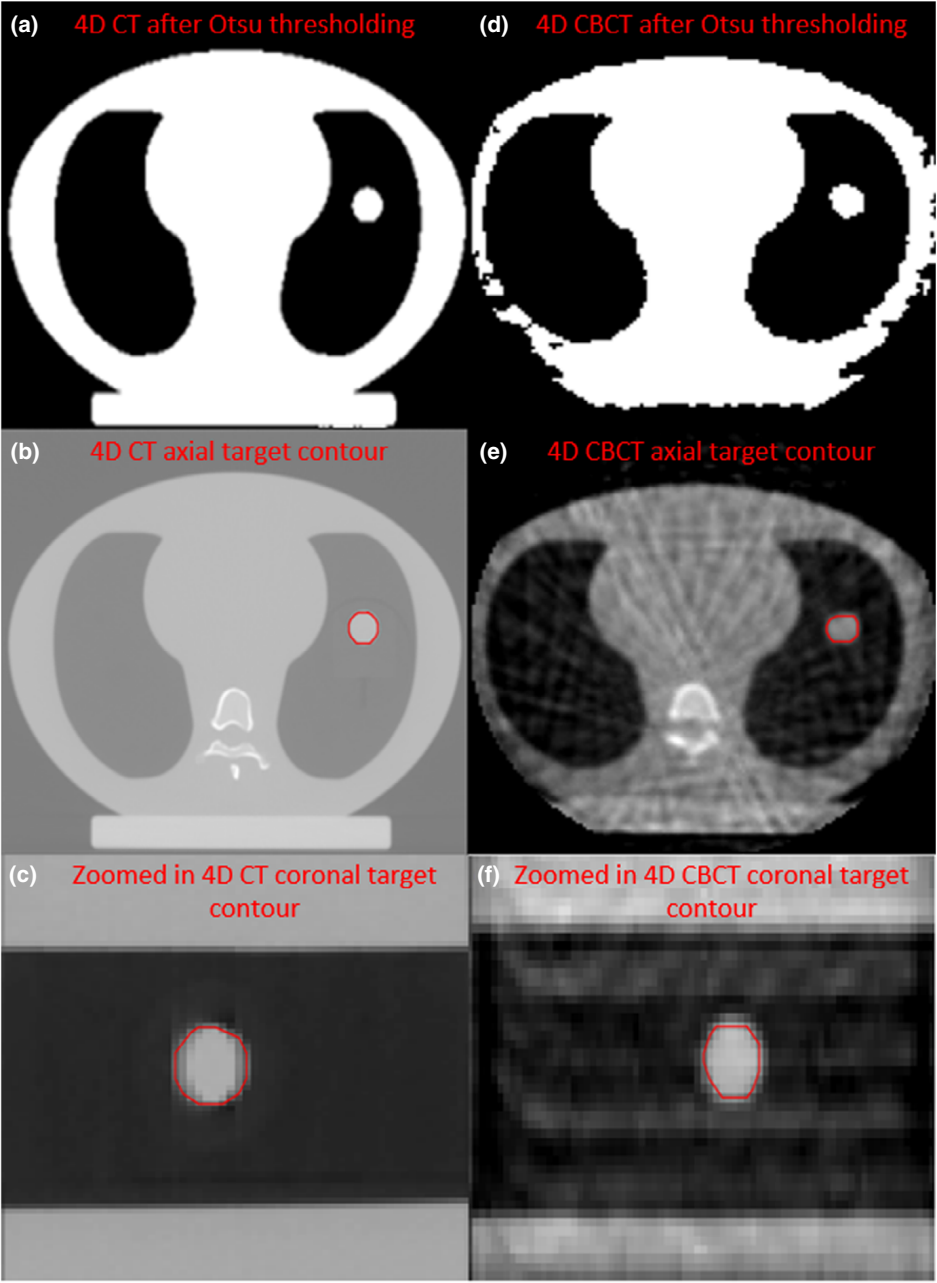
Illustration of binarizing four‐dimensional computer tomography (4D CT) (a–c) and four‐dimensional cone‐beam computer tomography (4D CBCT) (d–f) images to find the target and visually inspecting contours in the axial (b,e) and coronal (c,f) planes.

The progression of binarizing the 4D data sets to visual inspection of the target being properly found by segmentation in the axial and coronal planes is illustrated in Fig. 3.

**Fig. 4:**
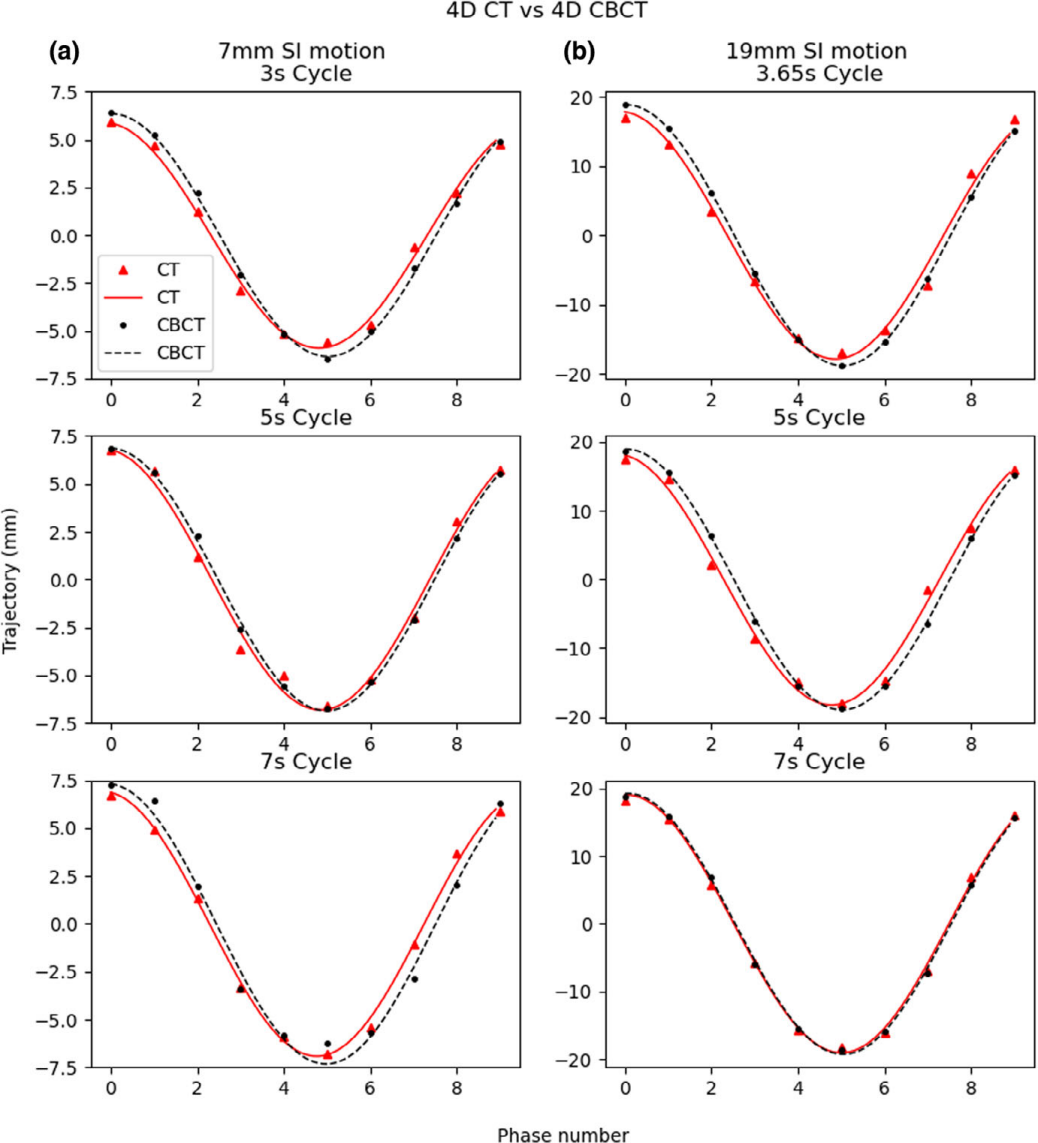
Sine curves fit to the trajectory of the target centroid for each phase. The red triangles and black circles represent calculated target trajectory with respect to phase for four‐dimensional computer tomography (4D CT) and four‐dimensional cone‐beam computer tomography (4D CBCT) data, respectively. Similarly, the red solid line demonstrates the sine curve fit for 4D CT data and the black dashed line for 4D CBCT data. Examples are shown for 7‐mm superior–inferior (SI) motion with cycle times of 3, 5, and 7 s (a) and 19‐mm SI motion with cycle times of 3.65, 5, and 7 s (b).

The programmed phantom motion parameters and corresponding amplitudes from sinusoidal curve fitting extracted from the 4D CT and 4D CBCT data sets appear in Table 1. The measured amplitude from visual inspection of the average 4D CT is shown in parenthesis. For the 3‐mm amplitude, cycle times of 5 and 7 s resulted in severe 4D CBCT artifacts, leaving image analysis not possible. The 5‐mm amplitude, 7‐s cycle time scenario encountered the same issue. Additionally, the 11‐, 13‐, and 15‐mm amplitude 3‐s cycle times experienced motion artifacts in the 4D CT that were significant, and image processing for curve fitting was inappropriate. With regard to the 17‐ and 19‐mm amplitudes, a 3‐s cycle time was not programmable due to phantom speed limitation, and the lowest possible programmable cycle time was used instead, which resulted in 3.35 and 3.65 s for 17 and 19 mm, respectively. Differences between curve fitted data were smaller than 1 mm with the exception of one programmed motion resulting in a difference of 1.05 mm between the curve fits at 15‐mm amplitude and 7‐s cycle. Figure 4 shows an example of the resulting sinusoidal curve fitted graphs for the three cycles times explored in the 7‐ and 19‐mm amplitude cases.

**Table 1:** Programmed parameters of the CIRS dynamic thorax motion phantom with amplitudes from curve fits using 4D CT and 4D CBCT. The value in red indicates an abolsute difference greater than 1 mm.

Programmed amplitude (mm)	Cycle time (s)	4D CT curve fit amplitude (mm)	4D CBCT curve fit amplitude (mm)	Absolute difference between curve fits (mm)
**3**	3	2.56 (2.60)	1.63	0.93
	5	‐	‐	‐
	7	‐	‐	‐
**5**	3	4.19 (4.98)	4.14	0.05
	5	4.82 (5.14)	4.71	0.11
	7	‐	‐	‐
**7**	3	5.91 (6.79)	6.35	0.44
	5	6.92 (7.48)	6.87	0.05
	7	6.85 (7.19)	7.52	0.67
**9**	3	8.14 (8.83)	8.69	0.55
	5	8.66 (8.79)	8.93	0.27
	7	9.22 (9.31)	9.04	0.18
**11**	3	(10.64)	10.89	‐
	5	10.31 (10.40)	11.14	0.83
	7	10.71 (10.89)	11.12	0.41
**13**	3	(12.28)	13.10	‐
	5	12.55 (12.39)	13.14	0.59
	7	13.69 (12.52)	13.18	0.51
**15**	3	(14.36)	15.07	‐
	5	14.49 (14.50)	15.21	0.72
	7	14.10 (14.96)	15.15	1.05
**17**	3.35	16.01 (16.16)	16.79	0.78
	5	16.36 (16.37)	16.86	0.50
	7	17.01 (16.61)	17.34	0.33
**19**	3.65	17.89 (18.25)	18.83	0.94
	5	18.18 (18.15)	18.92	0.74
	7	18.96 (18.40)	19.24	0.28

Note

Values in parenthesis represent amplitude measured from visual inspection of the average 4D CT.

Abbreviations: 4D CBCT, four‐dimensional cone‐beam computer tomography; 4D CT, four‐dimensional computer tomography.

**Fig. 5:**
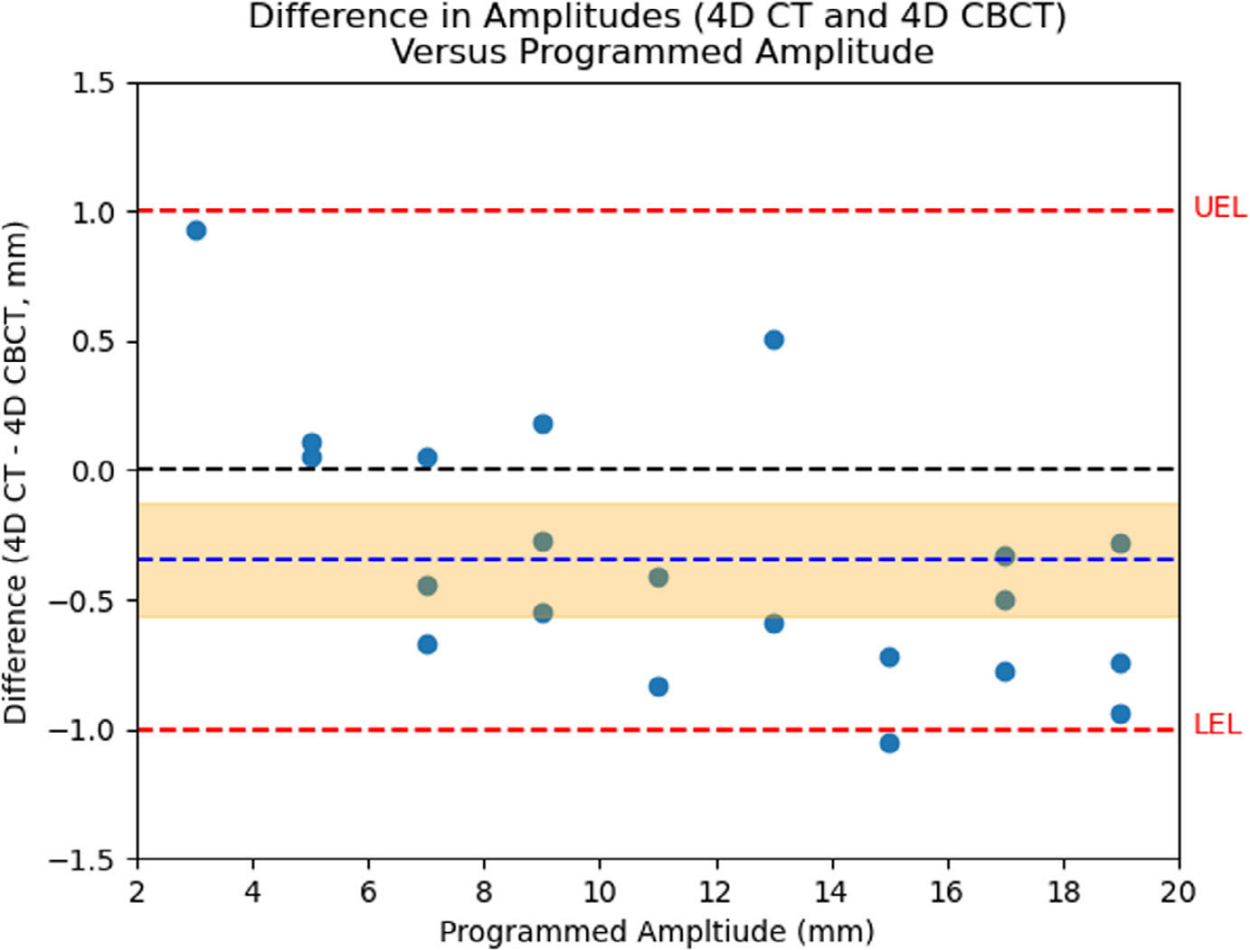
Scatter plot of the difference in amplitudes between four‐dimensional computer tomography (4D CT) and four‐dimensional cone‐beam computer tomography (4D CBCT) versus programmed amplitude. The mean difference is shown as a blue line, and its confidence interval is shaded around it in orange. The red lines illustrate the upper and lower equivalence limits.

A normal distribution of the difference between curve fitted data points was determined using the Shapiro–Wilk test (*p =*0.18). The mean difference between the two modalities was −0.35 ± 0.22 mm, representing a small but significant bias of 4D CBCT overestimating amplitude with respect to 4D CT. Additionally, root‐mean‐square‐error was determined to be 0.60 mm. The two imaging methods were determined to be significantly equivalent within this interval (*p* < 0.001) based on the two TOSTs used. The amplitude difference between measurements versus the programmed amplitude, equivalence limits, and mean difference with error is shown in Fig. 5.

**Fig. 6:**
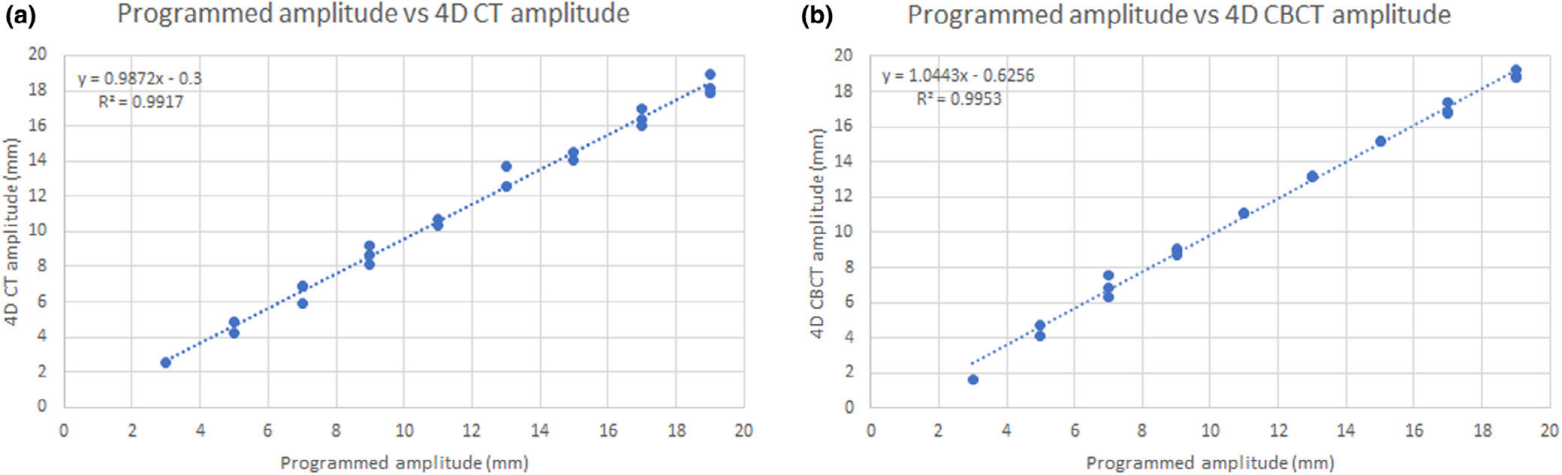
Plots of linear regression for programmed amplitudes versus four‐dimensional computer tomography (4D CT) (a) and four‐dimensional cone‐beam computer tomography (4D CBCT) (b) amplitudes.

The correlation coefficients between the true amplitudes programmed at the phantom and curve fitted amplitudes of 4D CT and 4D CBCT were 0.9958 and 0.9976, respectively. Similarly, the coefficient of determination (*R* ^2^) was 0.9917 for 4D CT and 0.9953 for 4D CBCT. Figure 6 shows the measured amplitude from curve fitting versus the programmed amplitude for 4D CT and 4D CBCT with linear regression.

### 3.2 Patient waveforms

The target size, measured amplitudes, and difference in amplitudes between 4D CT and 4D CBCT for simulated patient respiratory motion are shown in Table 2. The mean difference was 1.5 ± 2.1, 0.4 ± 0.3, and 0.8 ± 0.6 mm for SI, AP, and LR, respectively. In the SI direction, the mean difference was 0.8 ± 0.6 with the exclusion of the outlier shown in Table 2 for Patient 3.

**Table 2:** Target size, measured amplitude, and difference in amplitude between 4D CT and 4D CBCT with respect to patient number. Values in red indicate an absolute difference greater than 1 mm.

Patient	Target diameter (cm)	SI amplitude			AP amplitude			LR amplitude		
		4D CT (mm)	4D CBCT (mm)	Absolute difference (mm)	4D CT (mm)	4D CBCT (mm)	Absolute difference (mm)	4D CT (mm)	4D CBCT (mm)	Absolute difference (mm)
1	3	2.8	2.0	0.8	2.8	2.5	0.3	1.0	0.5	0.5
2	3	4.0	4.1	0.1	1.1	1.5	0.4	0.7	0.8	0.2
3	1	5.5	11.6	6.2	1.4	0.9	0.5	1.2	0.6	0.7
4	1	6.0	5.0	1.0	1.9	1.0	0.9	2.1	0.6	1.5
5	3	1.5	1.6	0.1	2.2	2.4	0.3	1.6	1.3	0.3
6	2	0.9	2.4	1.6	1.8	1.8	0.1	1.1	1.7	0.6
7	3	2.1	3.1	1.0	2.7	2.9	0.3	2.9	1.2	1.7

Abbreviations: 4D CBCT, four‐dimensional cone‐beam computer tomography; 4D CT, four‐dimensional computer tomography; AP, anterior–posterior; LR, left–right; SI, superior–inferior.

## 4 DISCUSSION

The application of 4D CT is considered standard protocol in SBRT treatment planning and simulation of mobile lung tumors. An average CT and maximum intensity projection derived from the 4D CT are often used for these purposes. This allows for visualization of the entire range of tumor motion and thus allows for patient‐specific motion management. Due to the nature of SBRT with high doses per fraction and steep dose gradients, it is paramount that localization be as accurate as possible to the 4D CT information used for treatment planning. A method to increase confidence in target localization at the time of treatment is utilization of 4D CBCT. Analogous to 4D CT, 4D CBCT allows for visualization of tumor motion throughout the respiratory cycle showing changes in the tumor trajectory between and/or immediately prior to treatments. Because the 4D CBCT used for localization is registered to the 4D CT data used for treatment planning, it is important to determine if these two imaging modalities agree with one another in terms of target motion in a controlled setting.

In this study, equivalence between 4D CT and 4D CBCT in the absence of patient related factors was explored by collecting 4D data sets of sinusoidal motion with varying amplitudes and cycle times. There was a measurable bias, too small to be clinically meaningful, where the 4D CBCT overestimated the amplitude with respect the 4D CT. However, 4D CBCT appeared to be closer to the ground truth value programmed for the phantom. The better accuracy associated with 4D CBCT is likely due to the presence of motion artifacts in 4D CT and the difference in slice thickness and image quality between the two modalities resulting primarily from difference in image acquisition (multislice in CT vs. volumetric in CBCT). Previous research has shown and sought to reconcile the fact that at large amplitudes and fast respiratory cycles, the presence of motion artifacts in 4D CT data becomes more pronounced.^31^, ^32^ Additionally, the slice thickness for 4D CBCT was finer than 4D CT at 2 and 2.5 mm, respectively. These slice thicknesses were chosen because we wanted the results to reflect what is the most clinically relevant scenario. As for the equivalence testing, the two imaging modalities were equivalent within the −1‐ to 1‐mm limits when severe artifacts were not present. The a priori difference interval of ±1 mm was selected based on what error is generally acceptable for SBRT treatments.

Following the controlled analysis of sine motion, patient respiratory waveforms were evaluated to see how the modalities agreed with more realistic patient motion, which includes occasional breathing irregularities. The error between the two modalities increased as expected intuitively due to the variation in patient breathing, but all values remained within 2 mm of each other except for Patient 3 whose SI amplitude was severely underestimated by 4D CT. It is suspected that 4D CT may be more subject to breathing irregularities at table positions because it only spends the specified cine time at each, while 4D CBCT is less impacted due to its continuous volumetric imaging method.

For the limitations of this study, a few of the small sine amplitude, slow cycle time combinations could not be analyzed due to 4D CBCT artifacts thought to manifest from inappropriate binning. Without being able to analyze these data sets, it is difficult to know how 4D CBCT may behave at similar motion parameters in a patient. Several 4D CT motion artifacts were also seen as the amplitude became large and cycle time decreased. This led us to explore the amplitude through observation of the average 4D CT, which is less objective than the curve fitting method. Another limitation was that some of the large amplitudes were physically constrained as to the minimum cycle times allowable by the software, requiring additional cycle time to be programmed. It is probable that the added cycle time reduced motion artifacts for these large amplitudes, and if they were not limited by programmable time constraints, the 4D CT trend of motion artifacts would have persisted. Lastly, in the evaluation of sine motion, only the SI direction was examined in this study. One motion combination of 10‐mm SI, 5‐mm AP, and 5‐s cycle was explored to evaluate motion in a combination of planes. This resulted in curve fitting 9.69 mm in the SI direction and 4.76 mm in the AP direction for the 4D CT. Similarly, 10.05 and 5.04 mm were determined from the SI and AP motion, respectively, for 4D CBCT. Additional AP and LR motion was not explored because previous research has shown that motion is most significant in amplitude in the SI direction.^24^ The resolution is also better in LR and AP directions versus the SI direction so resolving motion in the SI direction is likely the primary limitation. This can also be deduced from the AP and LR motion from the patient waveform evaluations that had a maximum difference of 1.7 mm. If this is the maximum error in the case of cycle variability due to breathing, it is unlikely to measure motion worse than the SI direction.

In the case of mobile tumors and free breathing, 4D CBCT has already proven to reduce the uncertainty of tumor motion when compared with standard 3D CBCT protocols.^7^, ^13^ The novelty of this work, however, is in understanding if 4D CT and 4D CBCT agree in their assessment of target trajectory over a wide range of motion parameters in a controlled setting. The controlled setting allows us to determine if the two modalities are inherently similar without additional variability of patient factors. Simulation and treatment planning are based on 4D CT data. It is therefore important that the 4D CBCT not only be accurate with respect to true target motion but also be consistent with the amplitude measured by the 4D CT as this is the information that registration is subject to. It was shown in this study that 4D CBCT is equivalent to 4D CT within millimeter accuracy in the presence of regular sinusoidal motion. However, in the presence of simulated patient breathing motion, the error increased but not beyond 1.7 mm with the exception of an outlier. For patients who may be coached during imaging and are unlikely to vary greatly between respiratory cycles, providers should have confidence in localization accuracy of the imaging modalities themselves in the case of SBRT treatments. Where coaching is not possible, appropriate choice of a setup margin can correct for these uncertainties. Modalities such as 4D CBCT can confirm that PTV volumes are inclusive of changes in breathing. Furthermore, 4D CBCT suffers less from motion artifacts and has a strong correlation to the true regular sinusoidal motion amplitude. Previous studies have also demonstrated that 4D CBCT accurately assesses target volume.^21^ It may also be useful to include 4D CBCT imaging in the treatment planning process for conventional fractionation to more accurately determine ITV, where clinics lack 4D CT capabilities but have 4D CBCT.

## 5 CONCLUSION

A thorax motion phantom was used to simulate target sinusoidal and simulated patient respiratory motion to evaluate target motion measured by 4D CT and 4D CBCT. The sine motion amplitudes were measured by sinusoidal curve fits, and the patient waveform motion was measured by segmentation of the target using the average 4D data sets. The two methods were equivalent within a 1‐mm limit for sine motion, and the error did not exceed 2 mm in the case of patient waveform motion with the exception of a single outlier. In cases where 4D CT is used to image mobile tumors for simulation and treatment planning, 4D CBCT will evaluate motion within at least 2‐mm accuracy of 4D CT in the absence of severe artifacts or changes in respiration between simulation and treatment, making it an attractive localization method due to its reduced motion artifacts and capability to visualize daily breathing motion. This is particularly applicable to lung SBRT treatments where less fractions and imaging are required, but the accuracy of treatment delivery is paramount. Any discrepancies between the motion observed at simulation and time of treatment may therefore be attributed to patient‐related factors such as setup, significant breathing irregularity, and changing target trajectory.

## CONFLICT OF INTEREST

Authors declare no conflicts of interest.

## AUTHOR CONTRIBUTIONS

C.B. and D.S. designed the project with S.S., T.W., and N.P. contributing to the concept. N.K., P.M., and K. R. contributed with data analysis and interpretation. C.B. and D.S. prepared the manuscript where all authors revised and edited the draft.

## Data Availability

The data that support the findings of this study are available from the corresponding author upon reasonable request.
